# Medical Items and News of Interest to the Physician

**Published:** 1878-01

**Authors:** 


### For the Use of Physicians.

CULLED FROM THE STANDARD MEDICAL JOURNALS OF THE WORLD.

NOTE.—These formulæ are intended only for the regular practitioner of medicine, and should be employed only by him, or under his immediate supervision.

**Auto-Transfusion.**

This is the title applied to the process of forcing the blood to the viscera by applying an elastic bandage to the limbs. It is easily done, attended with no risks, and an admirable auxiliary to treatment in post-partum hemorrhage and other maladies.—*Med. and Surg. Reporter, November 24th.*

**Chronic Cystitis.**

Dr. Mulhorn reported, before the late meeting of the Wayne County Medical Society, Michigan, that he had a lady patient who suffered from cystitis for three years. There was frequent desire to urinate, but ten-grain doses of benzoic acid very promptly relieved this difficulty. He has found benzoic acid to work like a charm in cystitis.—*Med. and Surg. Rep.*

**Diarrhœa in Children.**

Dr. L. A. Davidson of West Virginia sends the following as a most efficacious prescription for diarrhœa in children:

℞

Pulv. calumbæ. ℈ ij.—iv.

Ext. valerian fluid.

Syr. rhei, āā fl. ℥ ss.

M.—A teaspoonful every two hours.

**Chronic Diarrhœa.**

Dr. Bonamy (*Bull. Gen. de Therap*) has employed oxide of zinc with great success in obstinate diarrhœa characterized by the frequent passage of abundant sero-bilious stools, without tenesmus or colic, and with only moderate pain in the abdomen. To prevent nausea and vomiting, he combines the zinc with carbonate of sodium:

℞
Zinci oxidi, gr. c.
Sodii bicarb., gr. xvj. M.

Div. in chart. no. vi. One to be taken every three hours.

In M. Bonamy's experience even the most rebellious diarrhœas, which had resisted all other means of treatment, yielded to this, and its effect was very rapid.—*Medical Times.*

**Belladonna in Dysentery.**

Dr. Smith, of Cloverdale, Cal., recommends the use of belladonna in the treatment of dysentery, and states that he has obtained more satisfactory results with it than with any other drug. He frequently gives from two to four drops of the fluid extract every one, two or three hours, until the griping pains are relieved. The full remedial effect is, in some cases, not manifest until slight delirium or disturbance of vision is produced, but these symptoms disappear when the belladonna has been withheld for a few hours.—*Nashville Journal of Med. and Surg., August*, 1877.

**Coco as a Remedy for Drowsiness.**

Noticing your article in the October number on Erythroxylon Coca, I thought perhaps the following might be of interest: In this vicinity lives a young lady about twenty-four years of age, who has been subject to fits from infancy. A short time ago she was taken with stupor or drowsiness. She could hardly be kept awake long enough to take her meals. Fluid Extract of Coca was prescribed in teaspoonfull doses. The patient began to recover from the first and grew rapidly better, until at the end of a week her drowsiness had entirely left her and has not occurred since, although it is more than three weeks since the medicine was discontinued.

**Phosphorus Pill.**

In the *British Medical Journal* Mr. William Martindale gives the following directions for making pills of phosphorus with the oil of theobroma, which he recommends to be used when patients can not be got to take phosphorized almond oil. One per cent. of phosphorus may be combined in the following way: Having melted the oil contained in a wide mouthed bottle, placed in a water bath, add the phosphorus, and, partly closing the mouth of the bottle, heat till this, too, melts, and the temperature of the mixture becomes about 180° Fahr.; then cork it tightly, and with a little brisk agitation the phosphorus will dissolve almost immediately. Allow the fluid to cool and solidify; and having in this condition divided it into suitable lots for rolling, beat each in a mortar before applying it to the machine, and work off quickly. A three-grain pill will contain one-thirty-third of a grain of phosphorus. They may be coated with a solution of sandarac in absolute alcohol in the following manner: Place the pills in a covered pot and pour upon them a few drops of the solution; agitate well, and turn them out upon a slab; separate them from each other, and allow them to dry in the air.

**Catarrh of the Bladder.**

Edlefsen recommends the use of chlorate of potassium in acute, as well as chronic vesical catarrh. It rapidly diminishes the proportion of pus in the urine, lessens, or even removes, the subjective troubles, and keeps the urine acid.

Edlefsen recommends this remedy in these cases more particularly when oil of turpentine, usually a very efficient remedy, is contra-indicated; thus, in co-existing catarrh of the stomach, gastric ulcer, or nephritis, he prescribes ol. terebinth, rectif., in doses of ten drops three or four times a day in capsules. The chlorate of potassium he prescribes in solution—fifteen to three hundred parts water; a tablespoonful in water every two or three hours.

---

**Tænium Solium in a Child Two Years of Age.**

Dr. D. W. Heisberg, of Nebraska city, writes to the *Medical Record:* According to medical authorities, tænia occurs very infrequently in early life. Dr. J. Lewis Smith, who has had much experience, says, in his work on 'Diseases of Children,' that he has met but one case only under the age of five years. Others have occurred, but are quite rare. I have now in my possession a specimen of tænia solium recently expelled from a child about two years of age. One portion thereof was fifty-nine inches long at the time of expulsion. It was accompanied by forty-nine detached and different segments. The whole length was estimated to be about eight feet. This was expelled after administering spts. terebinthinæ with ol. ricini. The tænia still remains however, as segments have since been discharged. I report the case from the fact that it occurs so seldom at this youthful age.—*Med. and Surg. Reporter.*

---

**The Cockroach in Dropsy.**

In Russia the common cockroach (*blatta orientalis*) is a favorite popular remedy for dropsy. Dr. P. Bogomolow, of St. Petersburgh, has lately examined its effects in nine cases of Bright's disease, heart disease, and other affections accompanied with severe dropsy, and in all the result was the same. There was an increase in the secretion of the urine and perspiration, with rapid disappearance of œdema, and also almost complete disappearance from the urine of albumen and renal derivatives. The dose was five to ten grains of the powdered cockroaches in the twenty-four hours, but they were also administered as a tincture. These insects do not, like cantharides, says the *Boston Journal of Chemistry*, produce any irritant action on the kidneys. Dr. Bogomolow has succeeded in extracting from them a crystalline body which he calls antihydrophin, and which is their active principle.

---

**Quantitative Estimation of Urea in Blood.**

Equal weights of blood and of crystallized sulphate of soda are boiled together, with constant stirring. Sufficient water is then added to replace the loss of weight which has been caused by evaporation. The whole is then filtered, and a portion of the filtrate, equal in weight to that of the blood originally employed, is taken. This is transferred to an apparatus from which the carbonic acid of the atmosphere has been carefully removed, and is boiled with hydrochloric acid. The resulting fumes are conducted through a closed vessel containing baryta water. A portion of this baryta is converted into carbonate by the carbonic acid resulting from the disintegration of the urea, and it is found that each cubic centimetre of carbonic acid thus evolved represents 0.002683 gramme of urea.—*Pharmaceut. Centralhalle.*

---

**Germs in Disease.**

According to observations reported to the French Academy, M. M. Pasteur and Joubert show that the terrible animal disease which is known as *charbon or sang de rate* (carbuncular gangrene), is caused by microscopic bacteridia, which were first observed by Dr. Davaigue, in 1850. It may therefore be classed with trichinosis and the itch, as a parasitic disorder. Vibrios, bacteria and bactoridia, are all found under two essentially distinct forms; either in translucent threads of variable length, multiplying rapidly by division, or in groups of little brilliant corpuscles formed in the interior of these threads, which separate from the parent, and constitute an apparently inert mass of points, from which countless legions of filiform individuals may come, having the same twofold methods of reproduction. The threads may be killed by drying, or by a heat much below that of boiling water. The germs, when dry, withstand temperatures from 120° to 130° C, or, 248° to 266° F.—*Med. and Surg. Reporter.*

---

**Origin of Diphtheria.**

Dr. E. M. Snow says, in his report as Superintendent of Health, Providence, Rhode Island:

The evidence constantly accumulates in this city that diphtheria depends for its existence and prevalence upon foul air, arising from local conditions of filth. Though it is inoculable, and in that way contagious, and is, perhaps, to some extent, infectious, there is no evidence that, practically, the disease is propagated by inoculation, contagion or infection. We may expect that diphtheria will continue through the winter, though perhaps with less severity than during the last two months; but it will still continue to search out its victims among those whose constitutions are prepared for it, by breathing foul air and other depressing causes. The necessity for disinfecting and removing offensive filth of every description continues as imperative as ever. Too much care cannot be taken in this respect.—*Med. and Surg. Reporter.*

**Dental Caries, and How to Prevent It.**

In a paper read before the British Medical Association, Alexander Stuart, F. R. C. S., maintains the view, now generally advocated by dental authors, that caries is due to the chemical action of acids. The chief source of acidity in the mouth, and the one to which must be ascribed the general prevalence of dental caries is, he maintains, the decomposition of food remaining in and between the teeth. This decomposition goes on rapidly so that if particles of food are allowed to remain in the mouth over night, the acid forms before morning. Assuming acidity to be the only proximate cause, and its neutralization the only prevention, Mr. Stuart urges the necessity of cleansing the teeth at night, and washing the mouth out with a solution of carbonate of soda in water, made agreeable by the addition of camphor, which latter is so inexpensive that it may be regularly used by all classes. The author expresses his firm conviction that were these simple means in general use, dental caries would be as rare as it is now common.—*Boston Medical Journal, September 1st,* 1877.

**Cracked Nipples.**

The success obtained by M. Cheron in the treatment of fissures of the anus with picric acid induced M. Charrier to use the same application in the treatment of fissures of the nipple. He found that it relieved the pain and checked the morbid secretions in a very short time, while at the same time the delicate epithelium of the nipple became, as it were, tanned, and much less susceptible to alteration. In seven patients the application brought about a complete cure in from six to twelve days. The pain was relieved in from twelve to twenty-four hours, and nursing could be resumed without inconvenience to either mother or child. It is necessary that the picric acid used should be chemically pure and completely deprived of soda. Two solutions are used, one containing 13 parts of acid to 1,000 of distilled water, and the other 1 part to the 1,000. The nipple should first be thoroughly cleansed with warm water and a fine sponge, and the concentrated solution then applied with a brush several times in succession to the fissures and all inflamed points. This is to be repeated every morning only, but after every nursing the nipple is to be held for three or four minutes in a small glass filled with the weaker solution.—*Gazette Medicale de Paris, August 4th.*

**Quinia in Small Doses in Malarial Diseases.**

A correspondent of the *American Practitioner* writes that Dr. Charles R. Greenleaf, U. S. A., gives the following formula as coming from an army surgeon, whose name and present residence were forgotten. The dose mentioned, it is claimed, will secure the full antiperiodic effects of the remedy, and prevent a recurrence of an attack of intermittent fever.

℞

Quiniæ sulphatis, gr. ij.
Acid. citrici, gr. iij.

Rub fine together and dissolve in

Aquæ, ℥ ss.
Potasse iodidi, gr. iij.
Aquæ, ℥ ss.

Mix the two solutions together, and administer the whole quantity at a dose.

**Sweet Spirit of Nitre a Solvent in Salicylic Acid.**

Dr. D. M. Barkley, Caseyville, Ky., writes to the *American Practitioner:* "As the administration of salicylic acid has become so extensive, and as a good solvent is desirable, I wish to make known, through the *Practicioner*, that spiritus nitri dulcis (sweet spirit of nitre) is the best solvent. I have been prescribing it for nearly two years in the treatment of malarial fevers, with uniform success; in many cases without the use of quinia. I employ this formula:

℞

Salicylic acid, ʒ j.

Sweet spirit of nitre, ℥ jv. M.

Sig.—One teaspoonful every two hours, for children; two to four teaspoonfuls for adults. It can be diluted with water if necessary, and can be combined with veratrum, gelsemium, aconite, etc. One ounce of nitre will dissolve sixteen grains of the pure acid, and make a clear solution. If that quantity of acid is increased, on the addition of water, the acid is precipitated—the mixture becomes semi-solid; add a little more of the nitre, and it becomes a clear solution again. I will add that in the treatment of intermittent and remittent fevers, salicylic acid is a remedy of great power and value, generally relieving those diseases within forty-eight hours.

---

SELECTED TREATMENTS FROM RECENT CLINICS.

**Tonic for Children.**

Professor Wallace considers the elixir of quinine, iron and strychnia one of the best tonics for children.

---

**Chafing in Infants.**

Perfect cleanliness; never use dry soiled diapers. Common starch (dry), sprinkled in water, may be applied after each passage. A wash may be used three times a day of this kind: Solution of hyposulphate of soda, five grains to the ounce of water, and after drying, anoint with cod liver oil, which has had added to it a little calomel and calamine.

---

**Chafing and Eczema of Vulva.**

℞

Calomel, ℥ ss.

Calaminæ, ℥ jss.

M.

Sig.—Apply several times a day.

---

**Functional Palpitation.**

Woman, age 43; palpitation every half-hour for months past.

℞

Tinc. digitalis, gtt. v.

Tinc. verat. viridis, gtt. ij.

Tinc. aconite, gt. i.

Tinc. zingiberis, gtt. xiij.

M.

Sig.—One dose, three times a day.

---

**Dropsy.**

℞

Pulv. scillæ,
Pulv. digitalis, aa gr. j.
Calomel, gr. ss.

M.

Sig.—One dose, twice a day.

---

**Use of Iron.**

Professor Meigs' method of using iron:

℞

Ferri. redas., gr. ij.

Sig.—One dose, taken shortly after each meal. If swallowed while the stomach is engaged in the act of digestion, it does not occasion any unpleasant sensations. It will generally cure anæmia in sixteen days.

**Chronic Pleurisy with Adhesions.**

℞

Pot. acetat., gr. xv.
Pot. iod., gr. v.
Syr. Tolu.,
Aquæ, āā ℨ j.

M.

Sig.—One dose, ter die.

---

**Muriate of Morphia**

Is said to be less apt to produce abscess than the sulphate in hypodemic use.

---

**Opium to Those With Whom it Disagrees.**

℞

Pulv. opii., gr. j.
Pot. carb., gr. x.

M.

Sig.—Dissolve in half a wineglass of water.

---

**Leucorrhœa.**

Saturated solution of chlorate of potash, used as an injection daily.

Also—

Saturated solution of acetate of lead, used once or twice a week, diluted with equal parts of water.

---

**Chilblains.**

℞

Collodion, ℨ ij.

Tr. ferri chloridi, ℨ ij.

Ol. ricini, gtt. v.

M.

Sig.—Paint with camel's hair pencil two or three times a day. A sure relief, and if applied in the earlier stages it will speedily cure.

---

**Triplex Pill.**

Aloes socotrinæ,

Scammon.,

Pil. hydrarg., aa ℥ i.

Ol. tiglii., gtt. xx.

Ol. carui, ℨ jss.

Tinc. aloes et myrrhæ, fl. ℨ ij.

M.

Ft. pil. No. CIV.

---

**Excellent Podophyllin Pill.**

℞

Podophyllin,
Ext. hyoscyami, aa gr. iij.
Saponis, gr. ivss.
Syrupi simp., gtt. vj.

M.

Div. in pil. No. xij.

*—Phil. Med. and Surg. Reporter.*

**Solutions of Quinia for Hypodermic Use.**

In his recent work on fevers, Professor Loomis gives the following formulæ for solutions of quinia for hypodermic use in remittent fevers :

"The solution of quinia, commonly employed by the English surgeons for this purpose, is made by adding 150 grs. of quinia and 50 drops of dilute hydrochloric acid to 4 oz. of water, and then evaporating the solution to 2 oz. Of this, 30 drops may be administered at each injection. Some add carbolic acid to a solution of quinia in dilute sulphuric acid. The carbolic acid is added to prevent abscess at the point where the injection is introduced.

"The formula of this solution is as follows :

℞

Quiniæ disulphatis, grs. i.
Acidi sulphurici, ♏ v.
Acid. carbolici, ♏ ij.
Aquæ distillat, ʒ j.

M.

Thirty minims is the quantity usually administered at each hypodermic injection ; it represents between three and four grains of quinia. I have recently used the following:

℞

Quiniæ sulph., ʒ j.

Acidi hydrobronici, ʒ ij.

Aquæ distillat, ʒ vi.

"Thirty minims contain 4 grains of quinia."

**Constipation and Hæmorrhoids.**

Dr. Rousselet, on the strength of forty-seven cases, confirms the favorable reports that have been given of the utility of podophyllin in habitual constipation. He prefers pills of the gum resin, and is of the opinion that the remedy is usually continued for too short a period, two or three months being required to contract the habit of passing a daily stool, which, too, should be always attempted at the same hour. He begins with one pill of a centigramme, adding another if necessary, and continues this dose daily for a fortnight ; after this he gives one every other day for a week, and increasing the interval by a day every week. If the bowels in the meantime act irregularly, he goes back to the daily dose again to diminish it. He prefers giving the pill with the last meal instead of at bedtime, as usually advised. Having observed hæmorrhoids, which so commonly accompany constipation disappear when this is relieved by the pills, Dr. Rousselet tried their effect on persons who suffered from hæmorrhoids as a substantive affection, giving one or two centigramme pills in the same way. His success has been very considerable, the stools becoming softened, and the pain, tumefaction, and vascular enlargement being promptly relieved. The remedy, however, may require to be continued daily for a month or two.—*Gaz. Med.*

**Dysmenorrhœa.**

In dysmenorrhœa of a rheumatic character, when the pain is severe and the flow scanty, N. S. Davis, of Chicago, uses the following :

℞

Tinct. cimicifugæ, ℥ iij.
Tinct. stramonii,
Vini colchici radicis, āā ℥ ss.

M.

Take one drachm at each mealtime in water. If by long continuance or unusual susceptibility, the cimicifuga causes dull headache, as is sometimes the case, either the dose may be lessened or fluid extract of cypripedium may be substituted. The colchicum may be lessened in proportion to the other ingredients, if the bowels are disturbed.

When the pain and soreness extends to the ovarian region, the following has been serviceable :

℞

Ammoniæ hydrochlor, ℥ iij.
Tinct stramonii, ℥ ss.
Tinct. cimicifugæ rac., ℥ iss.
Syr. glycyrrhizæ, ℥ ij.

M.

Take one teaspoonful at each mealtime in a tablespoonful of water.

In a recent case characterized by extremely painful menstruation accompanied by severe headache, continuing in less degree through the interval between periods, some constipation and slight feverishness, Dr. Davis ordered :

℞

Acid. salicylici, ʒ iij.

Sodæ bicarb, ʒ ij.

Tinct. stramonii,

Vin. colchici rad., āā ʒ iv.

Glycerinæ (puræ), ℥ i.

Aquæ, ℥ iij.

M.

Of this the patient took one teaspoonful before each mealtime and at bedtime in a little water. Three weeks after commencing the use of the remedy menstruation took place without pain, for the first time in several years. Bathing and other hygenic measures were also employed in this case.

**The Medical Value of Jaborandi.**

The *British Medical Journal* states that in the Highgate Sick Asylum, under the care of Dr. Dowse, special observations were made on the action of the newly introduced remedies, jaborandi, gelseminum, salicylic acid, etc.; and modern instruments, as the cardiograph and ophthalmoscope, were brought into daily requisition to elucidate complete diagnosis. Dr. Dowse said that his experience of the use of jaborandi did not lead him to think highly of its theraputic value when given alone, yet it was often of great use, when combined with other drugs, to promote elimination. In the following case of Bright's disease its efficacy was well shown: J. A., aged seventy-three; urine pale, gravity 1.010; albumen copious; hyaline casts. He had irritable heart trouble, some dyspnœa, and painful and swollen joints. Five grains of the extract were given three times a day with alkali and digitalis, producing profuse ptyalism and sweating. Under this treatment the joint soon became free from pain, the swelling of the limbs and the dyspnœa subsided, and he was rapidly improving in every way.

Dr. Dowes said he at one time thought that jaborandi would be found of value to reduce the night temperature of phthisis, but after a long experience he found such was not the case. He referred to its efficacy in promoting the flow of milk where the mammary gland was inactive after parturition, and remarked that its physiological action was of the greatest importance, especially when compared with the action of belladonna, to which it was in every way antagonistic. One curious feature relative to its action, which had not been noted by M. Vulpian, was the re-secretion of pilocarpine from the blood by the submaxillary gland. A patient who was taking it stated that if he re-swallowed the saliva produced by jaborandi, it then produced profuse sweating; whereas, on the other hand, if the saliva were ejected, only slight perspiration resulted.

### Experiment on the Development of the Tænia Solium in Man.

There has been considerable discussion over the question whether or not the cysticercus of man is identical with the cysticercus of the pig. If it be true that the cysticercus of man is the second phase of development of the tænia solium, the parasite will also be found in its perfect strobiline state in the intestines of men, and in all probability there alone. To settle this point, M. Redon of Lyons, swallowed in warm milk four cysts taken from the body of a dead man. As, however, it was possible that these cysterci had been derived from a tænia carried by some animal with which the man had been in frequent relation, M. Redon also administered a number of the cysts to some sucking pigs and dogs. The pigs all succumbed to enteritis, and at the autopsies the most careful examination failed to reveal any traces of the parasite. The dogs also did not contain any traces of the tapeworms. With M. Redon himself, however, the case was different. After an interval of three months and two days he found links of the worm in his passages. These were examined and found to belong to the tænia solium. Shortly afterwards an entire strobilus was passed, which will be placed in the medical museum at Lyons. These experiments settled the question of the nature and development of the cysticercus of man; they present, moreover, a striking exception to that apparently so absolute rule of the development of the parasites; that the same parasite cannot obtain its complete development in the same individual or in two individuals of the same species.—*Gazette Med. de Paris, Oct. 20th*, 1877.

### Chloride of Calcium.

Dr. Robert Bell recommends the chloride of calcium as a therapeutic agent which deserves a more complete recognition than it has of late received from the profession. Having used it extensively for the last four years, and having closely observed its effects, he feels justified in asserting that, in most forms of tubercular disease, and in the wasting diseases of childhood, whether tubercular or not, it is a remedy of inestimable value. It seems to possess a direct influence over assimilation, and Dr. Bell uses it in all cases in which the symptoms show a strict observance of dietetic rules. The remedy was put to a crucial test in the Cambridge Street Orphanage for Girls; here, the majority of girls, when admitted, showed unmistakable signs of tubercular disease; every case in which the drug was used improved, many of them rapidly, and the majority appear to have shaken off the disease entirely. In tubercular disease of the bones and joints of children, and in affections of the cervical glands, due to the same cause, Dr. Bell has never met with its equal. Several cases of pulmonary phthisis treated with this salt gave most gratifying results, especially in the early stages. The only case of tubercular peritonitis that came under Dr. Bell's notice, during the period of observation, yielded completely to the remedy. In conclusion, he says, that, as the medicine requires to be perseveringly used, the physician should not be discouraged by an apparent failure, but should give it a lengthened trial. Muriate of calcium can do no harm to the economy, and in many cases will be of incalculable service. In a communication to the Doctor, Sinclair Coghill speaks in equally high terms of the efficacy of this drug. Well known and highly esteemed up to fifty years ago, it has been completely crowded out by iodine and cod liver oil. The most convenient form of administration is a solution of the crystals of the salt in ℥viij.—xij. of distilled water, of which the dose for an adult is: ♏ xx—l. in milk or syrup.—*Lancet*, Aug. 25, 1877.

---

**Use of Carbonic Acid Gas at Vichy.**

Carbonic acid is employed at Vichy in the form of general and local baths, in douches and injections, and by the stomach. For the general baths ordinary bath tubs are used, which are simply covered by an impermeable cloth, designed to protect the head. The sittings last from twenty minutes to an hour. In the local bath the afflicted limbs are simply enclosed in cloth or caoutchouc bags, into which the gas is then introduced. These baths have been used, with more or less success, to relieve the pain of gout and rheumatism, of sciatic and other neuralgias, etc. In these cases the gas exerts an analgesic and a diaphoretic action. For douches and injections, the gas is conducted to the diseased parts by means of flexible caoutchouc tubes. These douches and injections have given excellent results in cases of prurites and spasm, in the different vaginal and uterine neuroses, which are so often the causes of sterility, and in ulceration of the cervix uteri. Most of the cases of simple ulceration heal rapidly under the treatment. The injections, however, must be used with some caution, for when the mucous membrane is inflamed and excoriated, a dangerous quantity of the gas may be obsorbed by the denuded surfaces The administration of the gas by the stomach does not seem to be of much use.

The analgesic and cicatrizing properties of carbonic acid have been demonstrated by the experiments of Surgeon Honsz and others. It relieves the pain of a blister almost immediately, and hastens the reformation of the cuticle. When the gas is injected into the bladder or vagina, a slight sensation of tickling and heat, which radiates towards the abdominal region, is felt at first, but it is soon followed by relief of the hyperæsthisias, and sometimes by complete cessation of the pains. Tollin has employed injections of carbonic acid gas in the treatment of cancerous ulcers of the cervix uteri. In two cases they relieved the pains completely and rapidly; in one the relief lasted twenty-four hours, in the other eight days. Of course the injections had no other influence on the general or local condition. In cases of rheumatic paralysis the carbonic acid baths seem to prove very useful in restoring the power of motion.—*Le Monoement Medical.*

**Bromium of Ammonium in Hay Fever.**

Dr. E. C. Seguin, of New York, reports (*Journal of Nervous and Mental Diseases*) that last year he induced two or three persons suffering from this disease to employ a strong gargle of bromide of ammonium, and to wash out the nasal passages with a weak solution of the same salt several times a day during the attack. The result was so gratifying that he is disposed to ask physicians to give a fair trial to this treatment during the coming summer and autumn. The gargle to be of the strength of ʒ j or ʒij of bromide to ℥ j water; the solution for the nares much weaker, of from 10 to 30 grains of bromide in ℥ j water.

---

**The Treatment of Neuralgia.**

The following cases and treatment of neuralgia are given by Mr. E. M. Boddy, in the *Medical Times and Gazette*, London:

CASE 1.—John S., aged fifty-five. Patient, a farmer, had been troubled with obstinate recurrent attacks of facial or definite neuralgia, and had tried various remedies, but had obtained no relief. He was in robust health, and there was nothing to be seen or felt on either the upper or lower jaw, and there were no decayed teeth. The attacks would come on violently and without any warning, and the pain was so excrutiating that he said it made him "feel mad." After taking the opium and arsenic he fell into a profound sleep, and on awakening the pain had entirely left him. Now, in this case, relief was almost immediately afforded, and no further treatment was necessary, for the attack, no doubt, depended upon "some obscure irritation of the fifth pair of nerves," and was not caused by the health being out of gear.

CASE 2.—Henry H., aged forty, had been suffering, for some weeks previous to my seeing him, from the most excrutiating attacks, and there were no carious teeth to account for them. As he was out of health, I treated it as a case of anæmic neuralgia, and so I put him on a course of quinine and iron, which gave him no relief. At last he had such a severe attack that he was like one bereft; but the pain was immediately alleviated by the opium and arsenic, and left him, he said, "like a miracle." I *now* recommended the tonic treatment, and he very soon regained his ordinary health. In this case the neuralgia simply resulted from an "obscure irritation of the fifth pair of nerves," accompanied with debility.

CASE 3.—Charlotte B., aged eighteen. Patient was what one might term in first-rate health, and, strange to say, had never had the toothache. One evening, without any assignable cause, she was attacked with the most "horrible pain" in the face; had never experienced it before. I administered the opium and arsenic, and the pain at once left her. The next day she had another attack, which immediately succumbed to the remedy.

CASE 4.—Annie H., aged twenty-two. Patient had been irregular from puberty, and for the last six years had been subject to facial neuralgia, and no remedy had afforded her the slightest relief. She was a well formed girl, but was decidedly chloratic and anæmic. The first dose relieved the pain slightly; the second entirely removed it, for she slept soundly, and there was no vestige of it on awaking. I now treated her general health by administering purgatives and tonics, such as iron and quinine. When I last saw her she had no recurrence of the neuralgia, the catamenia were regular, and her general health had greatly improved.

These four cases, which I have selected from many others of which I have notes, are quite sufficient to show the efficacy of the combination of opium and arsenic in the treatment of this disease. Three of them are very good specimens of what I call definite neuralgia, and the last was of the same kind, though partly owing its origin to some uterine derangement. There is one very noticeable fact—they all derived marked benefit from the remedy; it also quickly relieved them of an agonizing pain, prevented its return, and no ill consequences resulted; and what is greatly in its favor, the opium promotes rest, which is so necessary, and the sufferer awakes feeling almost a new being, especially if the pain has been of long continuance.

The form of definite neuralgia which arises from hysteria is also amenable to opium and arsenic; but then it is desirable to give the patient a nervine sedative, such as the bromide of potassium or the tincture of valarian, after the neuralgic pain has subsided. The following is the mixture I always give:

℞

Liq. arsen., ʒ ss.

Tinct. opii, ʒ jss.

Aquæ, ad ℥ iij.

M.

Sig.—One tablespoonful to be taken when required.

The strength may be increased in very violent cases, but I have generally found the above sufficiently strong.—*Med. and Surg. Reporter.*

---

**Smoking Arsenic in Phthisis Pulmonalis.**

The roving gypsies and horse-jockeys of most countries give arsenic as a remedy for broken wind and heaves to horses, and with astonishing success, improving the general condition of the animal, giving him a fine healthy skin and sleek coat, also removing the difficulty of breathing. The only difficulty with its use was, as they say, that once begun it must be continued. In these cases it seems to act by stimulating the secretions generally, especially that of the skin, and improving the digestive function. This practice has been found common among the Arabs and wandering Tartars.

The northern Chinese use arsenic daily, mixed with their smoking tobacco.

The publication of M. Monteguy's statements with respect to the Chinese arsenic smokers called forth a letter from a Dr. Londe, who announced that some years previous, in the course of a discussion at the Academy of Medicine, Paris, on the agents to be employed to cure tubercular consumption, he told the assembled doctors that he had found but one successful means of combatting the dreadful disease—that means was the smoking of arsenic. He reaffirmed his commendation of the remedy. Trousseau, than whom few are more eminent, recommends the inhalation of arsenic by means of cigarettes saturated in a solution containing from one-half to one drachm to the ounce of arseniate of soda in the treatment of phthisis pulmonalis. Trousseau recommended that sheets of white paper be dipped into a solution composed of *one* part of arseniate of soda and *thirty* of water. The paper was then made into cigarettes or little cigars, and the patient directed to smoke one or two daily, inhaling the fumes at the moment they entered the mouth. At first they caused a little irritation, but after a short time the cough and expectoration diminished.

In weak states of the system, as in the course of phthisis where dropsy of the cellular tissue supervenes, arsenic is found beneficial in removing the anasarca, apparently acting as a tissue stimulant. While not forgetting the dangers of an overdose of this remedy, we feel—from personal observation of its beneficial effects in lung troubles, including phthisis with emaciation, especially bronchial phthisis, spasmodic asthma, bronchitis and catarrhal affections when smoked in the form of the arsenious acid commingled with a just proportion of stramonium leaves and lobelia, with nitrate of potash to secure combustion—that it cannot be too highly recommended in the treatment of lung affections when its administration can be regulated by a competent physician.—*Canada Lancet.*

---

☞ Please send in your subscription *at once*, and avoid being stricken off the roll.

